# Datasets of textile fibre persistence and the influence of physical activity for forensic interpretation and statistical analysis

**DOI:** 10.1016/j.dib.2025.112228

**Published:** 2025-11-01

**Authors:** Victoria Lau, Claude Roux, Xanthe Spindler

**Affiliations:** University of Technology Sydney, Centre for Forensic Science, Australia

**Keywords:** Textile fibres, Persistence, Forensic science, Microtrace, Trace evidence, Activity, Time

## Abstract

The data presented support the research paper “The persistence of fibres following a choreographed assault: a quantitative assessment of the influence of physical activity”. These data describe features (generic fibre type, colour, location of recovery) of 175,948 fibres recovered from cotton T-shirts and polyester/cotton hoodies worn by participants maintaining an intensity of physical activity for an interval of up to four hours.

The dataset may be used to conduct further statistical analysis to compare or in combination with other studies examining variables implicated in trace persistence. Importantly, forensic science researchers and practitioners may use this data for education and for the evaluation of forensic fibre findings in casework.

Specifications Table

**Table:** 

Subject	Forensic Science, Statistics and probability.
Specific subject area	Microtraces, textile fibres*,* criminalistics
Type of data	Raw data: Table (XLSX).
Data collection	Subjects performed assault re-enactments to transfer fibres between a cotton T-shirt and polyester/cotton hoody as previously described [1]. Thereafter, a given intensity of physical activity (low, moderate, high) was maintained for a time interval (10, 30, 60, 120 or 240 min), before removing the garment. Garments were tapelifted to collect transferred and background fibres. All fibres in ten 1 × 1 cm grids per tapelift were counted and categorised by microscopic examination (Leica EZ4D and DM4M-FSCB) by colour and generic type. Donor garment fibres were defined as target fibres. Continuous length of a randomised subset of 5286 target fibres was measured (LAS V4.13.0) and categorised into four size ranges.
Data source location	University of Technology Sydney, Centre for Forensic Science, Ultimo, Sydney, Australia*.*
Data accessibility	Repository name: UTS Research Data Portal Data identification number: arcp://name,uts_public_data_repo/d3f2dd00db2011efb25da1b6db1b2a38 Direct URL to data: https://data.research.uts.edu.au/object?id=arcp%3A%2F%2Fname%2Cuts_public_data_repo%2Fd3f2dd00db2011efb25da1b6db1b2a38&_crateId=arcp%3A%2F%2Fname%2Cuts_public_data_repo%2Fd3f2dd00db2011efb25da1b6db1b2a38&fileId=persistence_raw.xlsx And https://data.research.uts.edu.au/object?id=arcp%3A%2F%2Fname%2Cuts_public_data_repo%2Fd3f2dd00db2011efb25da1b6db1b2a38&_crateId=arcp%3A%2F%2Fname%2Cuts_public_data_repo%2Fd3f2dd00db2011efb25da1b6db1b2a38&fileId=alldata_raw.xlsx
Related research article	*Lau, V., Roux, C., & Spindler, X. (2025). The persistence of fibres following a choreographed assault: A quantitative assessment of the influence of physical activity. Science & Justice.*https://doi.org/10.1016/j.scijus.2025.01.004.

## 1 Value of the Data

• The data shows the number, type, length and spatial distribution of textile fibres recovered from subjects performing physical activity for durations of up to 240 min. This provides valuable insight into the mechanisms and factors affecting the persistence of textile fibres under real-world conditions
• This contemporary data may be used to assist expert practitioners in interpretation, including the assignment of probabilities when calculating a likelihood ratio (LR) in the evaluation of forensic fibre findings. The data may also be used by researchers in future studies concerning fibres and other physical microtraces, the development of evaluative frameworks and in practitioner education and training.
• This is the first known published dataset of fibre persistence in which intensity of physical activity is a controlled variable; and record of the continuous length of target fibres recovered over time. Different quantitative and modelling approaches may be employed by researchers on this and data from similar studies to further investigate relationships on these properties and other measured variables.
• The novel experimental design and resultant data may be used to guide future simulation studies exploring other factors and physical traces.

## 2 Background

The persistence of textile fibres is a fundamental consideration in the interpretation of evidence in forensic science. Persistence is however influenced by numerous complex factors. Whilst prior research has advanced our understanding, much existing data originates from simulations that lack realism and scope for robust application in practice. Moreover, despite general recognition that activities of the wearer of a recipient substrate have an important role in persistence, empirical data to support this remains limited.

This dataset was acquired using human subjects in realistic conditions to quantitatively and qualitatively characterise the persistence of textile fibres transferred following a simulated assault [1]. The influence of wearer activity on fibre persistence over time was also examined. By providing contemporary real-world data, this dataset aims to support researchers and practitioners in evaluating fibre findings and addressing activity-level questions. Furthermore, it contributes to the broader disciplinary knowledge base, providing insight into mechanisms underlying the persistence of physical traces.

## 3 Data Description

The data herein extends on a publication which focused on the transfer of textile fibres between garments worn by participants re-enacting a frontal assault [2]. In this work, garment surfaces were tapelifted immediately upon conclusion of the simulated assault (T0) and examined to characterise the number, colour, generic type, length and zonal distribution of recovered fibres. The dataset from this study is publicly available [3] as a Microsoft Excel file.

The present dataset, accessible in [4], presents parameters of all recovered fibres examined and characterised following recovery up to 240 min after transfer (T10 – T240). In the repository, data for 175,949 fibres from all experimental replicates for the persistence study are given in the Excel workbook *persistence_raw.xlsx*, with one row corresponding to each individual fibre.

**Fig. 1:**
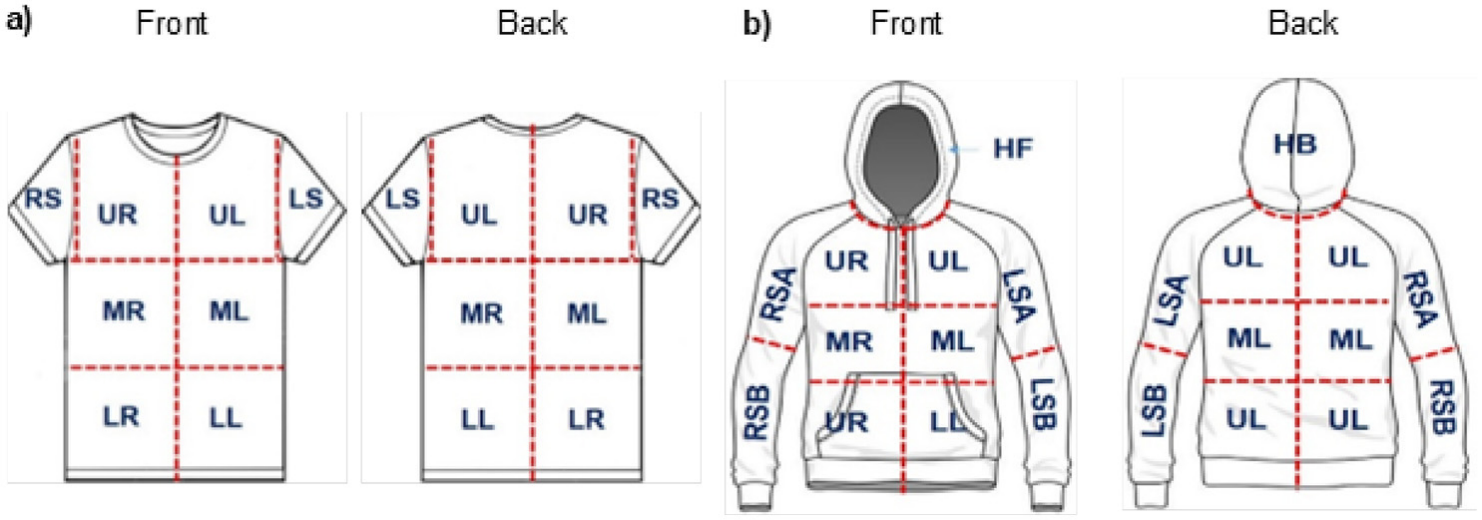
Diagram illustrating nomenclature and location of tapelifting zones on front and back surfaces of worn a) T-shirts (eight [8] zones), and b) hoodies (eleven [11] zones) each. Reproduced from [2].

The parameters specified are organised by: concatenated experimental code, experimental replicate (A to D), tapelifting zone, front (F) or back (B) surface of garment, lateral location (left [L], middle [M], right [R]), axial location (lower, middle, upper, sleeve, hood), garment size (XS, S, M, L), garment type (T-shirt [T], hoody [H]) and tapelifting grid (1 – 10). Subsequent columns specify characteristics of fibre colour, generic type, continuous length and categorical length. Fibre classification categories are detailed in Table 1; and the zonal regions depicted in Fig. 1. The last two columns indicate the duration of time the garment was worn in minutes (10, 30, 60, 120, 240); and intensity of physical activity (low, moderate, high).

**Table 1:** Classification of fibres recovered.

Property	Categories
Fibre colour	Black/grey (bk), blue (bl), dark blue (dbl), green (grn), orange/brown (org), other, purple (pur), red, turquoise (trq), yellow (ylw)
Fibre type	Cotton, linen/flax, other vegetable (veg), wool, hair, other animal (animal), man-made, miscellaneous (misc), unclassified
Continuous length	Numerical value (mm)
Categorical length	1 (≤ 1.0 mm), 2 (1.0–3.0 mm), 3 (3.1–5.0 mm), 4 (> 5.0 mm)

The Excel workbook *alldata_raw.xlsx* includes data from transfer experiments [3], in which T0 was considered control for intensity of physical activity. The data in individual worksheets correspond to each possible combination of activity intensity and time.

## 4 Experimental Design, Materials and Methods

### 4.1 Materials

Yellow cotton T-shirts (‘T-23 Base Tee’, 180 gsm 100% cotton jersey, Quoz, Revesby) and red polyester/cotton hoodies (‘Marshall Hoodie’, 310 gsm 80% cotton 20% polyester, Sportage Clothing Australia, Marrickville) were purchased from a wholesaler (F.M. Apparels, Northmead NSW, Australia) for use in the persistence study. Garments were separately laundered and blanked (cleaned) with a lint remover and adhesive tape prior to each experimental session.

48 mm adhesive tape (Scotch® Tough Grip Moving Tape, 3 M Australia) and clear acetate sheets (EXP600 OHP, Winc Australia Pty Ltd.) for fibre recovery were sourced from Officeworks Ltd. (Chadstone, Victoria).

### 4.2 Participants

21 jiu-jitsu practitioners of intermediate to advanced skill level (yellow to black belt) took part in the study. Each participant provided consent in all experiments approved by University of Technology Sydney Human Research Ethics Committee (HREC ETH18–3059).

### 4.3 Instrumentation

A Leica EZ4D stereoscopic zoom macroscope (Leica Microsystems GmbH, Wetzlar) equipped with an integrated digital 3.0-megapixel CMOS camera was used with brightfield and darkfield lighting for tapelift examination and morphological characterisation of recovered fibres. Leica Application Suite (LAS) software (v3.8, Leica Microsystems GmbH, Wetzlar) supplemented with the manual measurement add-on module (LAS V14.3.0) facilitated acquisition of digital micrographs and fibre length measurement. The DM4M-FSCB comparison microscope (Leica Microsystems GmbH, Wetzler) was used for further characterisation of target fibres at higher optical magnification (50 – 1000 ×).

### 4.4 Persistence experiments

A choreographed routine simulating an upper body-dominant assault between a ‘victim’ and ‘assailant’ was enacted by pairs of participants each wearing a yellow T-shirt or red hoody, respectively. Thereafter, participants continued to engage in physical activity of a low, moderate or high intensity level (Table 2) for a specified duration of time (10, 30, 60, 120 or 240 min) prior to garments being removed and packaged.

**Table 2:** Physical activity intensity levels and corresponding activities.

Activity Intensity	Activities
Low	Sedentary activities eg. watching TV, reading, computer work Easy walk, leisurely stroll, sitting in the train/bus/car
Moderate	Brisk walking, gentle bicycle riding, yoga Tasks often needing moderate effort and limb movement, eg. housework (laundry, sweeping)
High	Above activities with greater degree of body movement and/or effort Running, sprinting, jogging, hilly bicycle riding, vigorous aerobics Lifting, carrying, digging

### 4.5 Fibre recovery and characterisation

The front and back outer surfaces of garments were divided into zones (Fig. 1), each of which were sampled with a single length of adhesive tape using the “press and rub method” to recover fibres [5].

Each zonal tapelift was examined under low-powered microscopy (80 – 320×). All fibres in ten random 1 × 1 cm squares in each tape were examined, counted and classified according to colour. Those indistinguishable in colour and macroscopic morphology from transferred target fibres – yellow cotton (T-shirt) and red cotton and red man-made (hoody) – were marked for extraction and further classified according to generic type (Table 1). Digital micrographs were acquired of each grid. Preliminary classification of fibre type was further verified by examination of fibres mounted on glass slides under high-powered microscopy (50 – 1000x).

Continuous length measurement was performed on a proportional data subset. The sample population dataset was divided into 32 subgroups (strata) considering all combinations of garment type, physical activity intensity and time. 10% of each stratum was randomly sampled using the *sample_frac* function in R, creating a subpopulation of 5,286 target fibres (T10 – T240). The length of individual fibres was measured and recorded on the digital micrographs. Resultant continuous length values were tabulated in Microsoft Excel and categorised into the four size ranges (Table 1) using the VLOOKUP function.

## Limitations

Measurement of continuous length was limited to a stratified proportional subset (*n* = 5286) of the whole data population due to the labour-intensive nature of manual digital analysis and extensive size of the whole dataset. A greater sample proportion is warranted for comprehensive characterisation of recovered fibres.

## Ethics Statement

All human subjects were briefed to the research objectives and provided informed consent to participate in the research. All experiments were approved by the University of Technology Sydney Human Research Ethics Committee (HREC ETH18–3059).

## CRediT Author Statement

**Victoria Lau:** Conceptualisation, Methodology, Formal analysis, Investigation, Writing – original draft, Writing – review and editing, Visualisation. **Xanthe Spindler:** Conceptualisation, Supervision, Writing – review and editing, Project administration. **Claude Roux:** Conceptualisation, Supervision, Writing – review and editing, Project administration.

## Data Availability

UTS Research Data Portalalldata_raw.xlsx (Original data)
UTS Research Data Portalpersistence_raw.xlsx (Original data)
